# A preliminary investigation on the utility of temporal features of Force Myography in the two-class problem of grasp vs. no-grasp in the presence of upper-extremity movements

**DOI:** 10.1186/s12938-017-0349-4

**Published:** 2017-05-16

**Authors:** Gautam P. Sadarangani, Carlo Menon

**Affiliations:** Menrva Research Group, Schools of Mechatronic Systems and Engineering Science, Simon Fraser University, Metro Vancouver, BC Canada

**Keywords:** Activity monitoring, Force Myography, FMG, Stroke rehabilitation, Grasp detection, Wearable sensors

## Abstract

**Background:**

In upper-extremity stroke rehabilitation applications, the potential use of Force Myography (FMG) for detecting grasping is especially relevant, as the presence of grasping may be indicative of functional activity, which is a key goal of rehabilitation. To date, most FMG research has focused on the classification of the raw FMG signal (i.e. instantaneous FMG samples) in order to determine the state of the hand. However, given the temporal nature of force generation during grasping, the use of temporal feature extraction techniques may yield increased accuracy. In this study, the effectiveness of classifying temporal features of the FMG signal for the two-class grasp detection problem of “grasp” versus “no grasp” (i.e. no object in hand) was evaluated with ten healthy participants. The experimental protocol comprised grasp and move tasks, requiring the use of six different grasp types frequently used in daily living, in conjunction with arm and hand movements. Data corresponding to arm and hand movements without grasping were also included to evaluate robustness to false positives. The temporal features evaluated were mean absolute value (MAV), root mean squared (RMS), linear fit (LF), parabolic fit (PF), and autoregressive model (AR). Off-line classification performance of the five temporal features, with a 0.5 s extraction window, were determined and compared to that of the raw FMG signal using area under the receiver operating curve (AUC).

**Results:**

The raw FMG signal yielded AUC of 0.819 ± 0.098. LF and PF resulted in the greatest increases in classification performance, and provided statistically significant increases in performance. The largest increase obtained was with PF, yielding AUC of 0.869 ± 0.061, corresponding to a 6.1% relative increase over the raw FMG signal. Despite the additional fitting term provided by PF, classification performance did not significantly improve with PF when compared to LF.

**Conclusions:**

The results obtained indicate that temporal feature extraction techniques that derive models of the data within the window may yield modest improvements in FMG based grasp detection performance. In future studies, the use of model-based temporal features should be evaluated with FMG data from individuals with stroke, who might ultimately benefit from this technology.

## Background

Force Myography (FMG) involves measuring the outward force, or pressure, of a muscle as it changes conformation below the surface of the skin, as a means to characterize the state of the underlying musculo-tendinous complex [1]. The use of FMG in conjunction with machine learning and pattern recognition techniques is an active area of research [1–5]. FMG provides a simple to use, inexpensive, and unobtrusive method for detecting the functional state of a limb. Studies have shown that FMG can be used to (i) predict grip strength [1], (ii) predict single finger forces [2], (iii) detect hand gestures [3], (iv) detect grasp and move actions [4], and (v) detect upper-extremity postures, including grasping, elbow flexion, and wrist pronation [5]. Several applications have been proposed for FMG based sensing, including the creation of human machine interfaces to control robotic prosthesis [6] and industrial robots [7], as well as the creation of monitoring systems that encourage functional use of the limb as part of a stroke rehabilitation program [4, 5].

The potential use of FMG for grasp detection is especially relevant in the case of rehabilitation in individuals with upper-extremity impairments resulting, for instance, from stroke. Research suggests that it is necessary to practice hundreds, if not thousands, of grasp and release motions to optimize hand motor recovery after stroke [8, 9]. Accordingly, encouraging the use of the paretic limb to complete functional tasks in daily use is a key goal of stroke rehabilitation [10, 11]. In fact, given the wide variety of grasps required to complete activities of daily living (ADL) [12], the presence of grasping, regardless of the grasp type, may be indicative of functional use of a limb, which may contribute to hand motor recovery. The ability to distinguish between grasping, regardless of grasp type, versus no grasping (i.e. no object in hand) could allow FMG based devices to encourage stroke survivors to use their paretic arm functionally as part of daily living, and contribute towards improved rehabilitation outcomes.

Several studies with healthy participants have shown the feasibility of FMG based grasp detection [3, 4, 13, 14]. Despite the noted work, the use of FMG for grasp detection still presents challenges. In fact, the FMG signal has been shown to be sensitive to joint positions, including that of the elbow, forearm, and wrist [5, 15–17]. While the detection of upper-extremity joint positions may be useful in some applications, the sensitivity of FMG to joint positions could adversely impact grasp detection as joint positions will vary when the user is grasping and moving an object. Variations in joint position and movement trajectories, which would be expected as part of daily living, could confound the grasp detection classification scheme and reduce accuracy. Thus, further research is required to increase the robustness of FMG based grasp detection, such that it may be eventually used for grasp detection in unconstrained environments. Additionally, a majority of FMG classification research has focused on trying to distinguish between various types of grasps (i.e. multi-class problem) in the absence of significant upper-extremity movement [3, 18, 19]. The ability to distinguish between a discrete set of grasp-types is a necessary attribute in human machine interface applications [3, 18]. However, the presence of grasping, regardless of the grasp type involved, can be indicative of functional use of a limb, which is a key goal in stroke rehabilitation [11]. In stroke rehabilitation applications, FMG-based devices will have to detect and encourage grasping instead of a lack of grasping, regardless of which of the wide variety of grasp-types necessary to complete ADL [35], was used. To the best of our knowledge, there have been no studies that have evaluated the accuracy of FMG, or other upper-extremity sensing modalities, when classifying data associated with multiple grasp-types, in the presence of significant upper-extremity movements, for the two-class problem of grasping, regardless of grasp type, versus no grasping (i.e. no object in hand), which may be a more clinically relevant scenario.

Contemporary FMG research has focused on classification of the raw FMG signal, in the form of instantaneous FMG samples from multiple channels (i.e. sensors), in order to detect the grasping of an object [3–5, 13]. However, the grasping of an object is not a discrete, instantaneous action. Instead, it is a multi-stage process [20]. The force generated during grasping will increase as an individual first moves his or her fingers, makes contact with the object, forms the grasp, and then proceeds to generate force in order to be able to grip the object securely, and lift the object against gravity. Subsequently, the releasing process involves a reduction in force and the opening of the hand into a neutral hand posture [20]. Given that grasping involves a force profile that changes over the various stages of a grasp, we postulate that the temporal sequence of FMG values adjacent in time may provide descriptive information on the current grasping state of the hand (i.e. that grasp detection with FMG can be represented as a time series problem). Traditional machine learning methods, such as the support vector machine and neural network, use an instance based method for classification. In such methods, the instantaneous classifier output is dependent on the instantaneous inputs to the classifier; historical or future inputs do not affect the instantaneous classifier output. The use of temporal feature extraction has been shown to increase accuracy in applications that involve classifying data that have temporal or sequential dependence, such as the classification of electromyography (EMG) signals [21]. The temporal feature extraction step involves calculating parameters (i.e. features) that represent the time series within a certain window size of data. The calculated features are then provided to the classifier as instantaneous inputs [22].

Based on these considerations, the objective of this study is to evaluate the utility of classifying temporal features of the FMG signal for FMG data associated with a variety of grasp-types, in the presence of confounding upper-extremity movements, for the two-class grasp detection problem of grasp, regardless of grasp type, versus no grasp (i.e. no object in hand).

## Methods

### Participants

Healthy volunteers, with full upper-extremity functional ability, were recruited for the study. All participants provided informed consent for their participation in the study, which was approved by Office of Research Ethics, Simon Fraser University. Potential participants with height greater than allowed for by the experimental protocol were excluded from the study.

### Data collection device

**Fig. 1:**
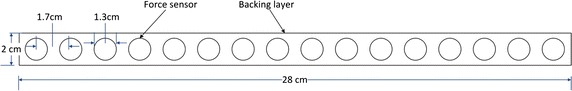
Internal dimensions of force sensing band

The experimental device consisted of a force sensing band with 16 force sensors and an additional external force sensor that was connected by flexible wire to the device’s housing. The force sensing band was 28 cm long and 2 cm wide; the center-to-center distance between successive force sensors on the band was 1.7 cm. Figure 1 depicts the dimensions of the force sensing band. The additional external force sensor was an FSR402 from Interlink electronics [23]. The 16 force sensors in the force sensing band were fabricated with a polymer thick film, which exhibits a reduction in resistance as force is applied to it. The sensing characteristics of sensors fabricated with this polymer thick film are the same as the FSR402 from Interlink electronics [23].

**Fig. 2:**
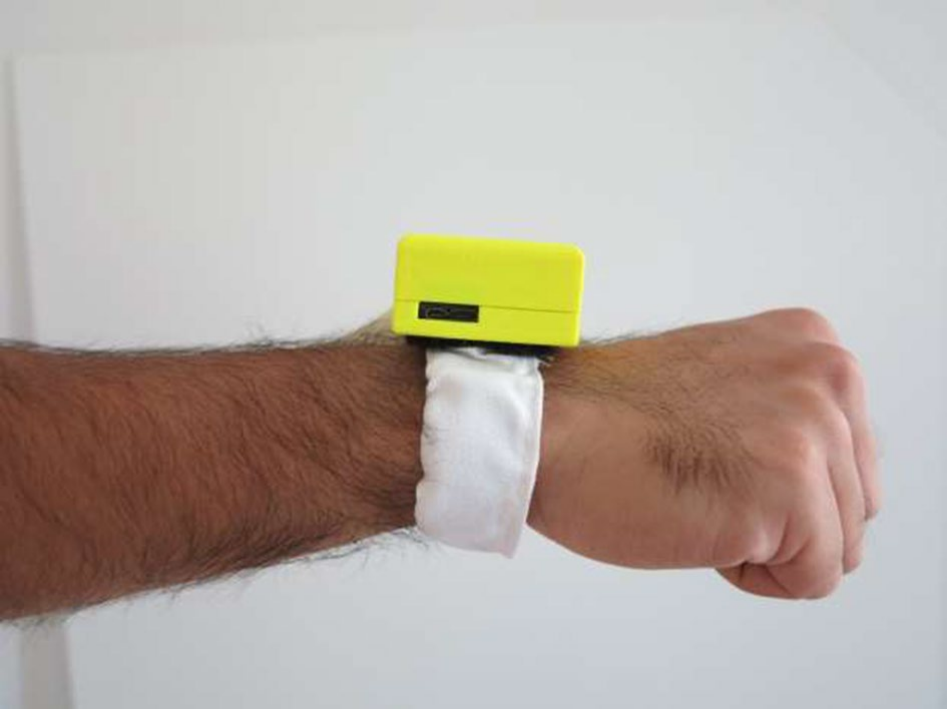
Device donning position

**Fig. 3:**
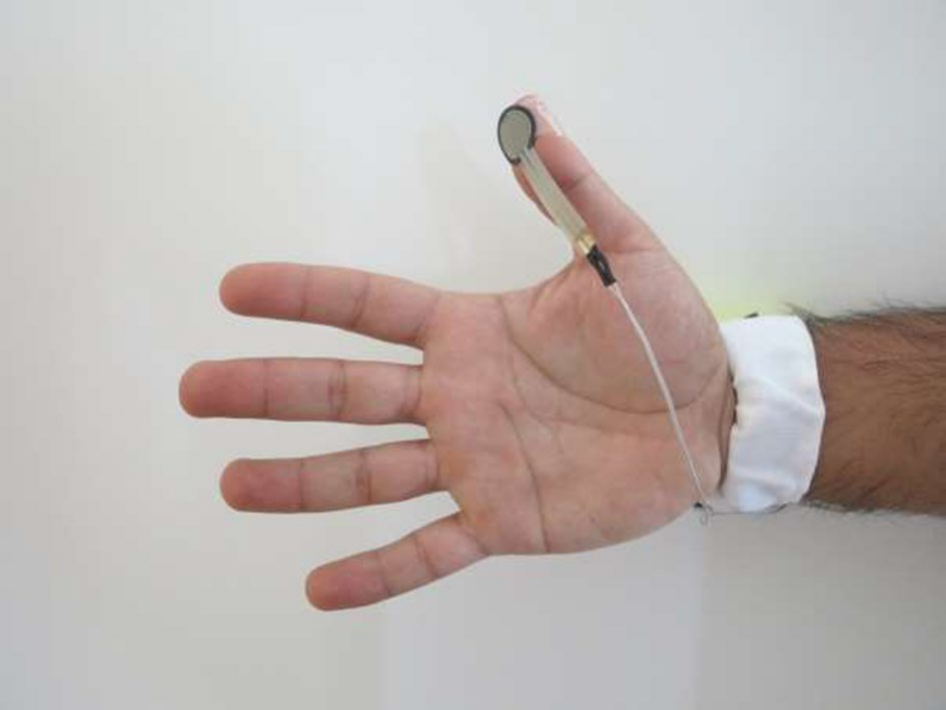
Thumb sensor for labelling data

The band was donned on the participant’s wrist, on the distal side of the styloid process of the Ulna bone (Fig. 2). In the event that the band was longer than the participant’s wrist circumference, the remaining length of the band was taped down to the device housing. The additional force sensor was taped to the participant’s thumb (i.e. thumb sensor). The thumb sensor, connected to the device housing, is depicted in Fig. 3. The signal from the thumb sensor was used to label each datum that corresponded to a grasp (i.e. whenever force was observed on the thumb sensor due to the grasping of an object) using a threshold crossing that was tuned by the experimenter. Data from the thumb sensor served as the true label for training classifiers and evaluating FMG classification performance. This method of labeling the data was motivated from previous works published by our research group [24] and was selected as all the grasp types used in this study involve active opposition of the thumb in order to form the grasp and secure an object.

The change in resistance demonstrated by each force sensor in the 16 channel (i.e. sensors) force sensing band, and the thumb sensor was quantified using a voltage divider circuit with a 20 kΩ resistor using a 10-bit analog to digital convertor. Data were sampled at 10 Hz with an AT Mega 328 Microcontroller, located within the device housing, and wirelessly transmitted to a Personal Computer via Bluetooth. The 10 Hz sampling rate has been shown to be sufficient for FMG based sensing and classification [4, 6, 25], as it has been shown that most volitional upper-extremity motion occurs below 4.5 Hz [26, 27]. Custom LabVIEW [28] software was written to collect data from the data acquisition device. The software consisted of a communications module that received and logged data from the FMG data acquisition device and a graphical user interface that allowed the operator to insert a label associated with each round of data collection in the experimental protocol.

### Experimental protocol

The experimental protocol comprised multiple repetitions of several grasp and move tasks. As noted previously, research has shown that the FMG signal is sensitive to joint positions of the wrist, forearm and elbow [5, 15], which are expected to vary with the varying movement trajectories that are likely to be employed when grasping and moving objects in daily use. In order to explore the limits of FMG grasp detection, the protocol was designed to involve movement in the three-dimensional workplace, and the use of joint movement (such as wrist flexion and extension, elbow flexion and extension, and shoulder flexion and extension) for task completion. Additionally, to evaluate the classifiers’ robustness to false positives (i.e. incorrectly detecting a grasp when no grasp occurred), portions of the protocol required movement without grasping (i.e. with a neutral hand posture) in the same three-dimensional workplace.

### Three-dimensional workspace

**Fig. 4:**
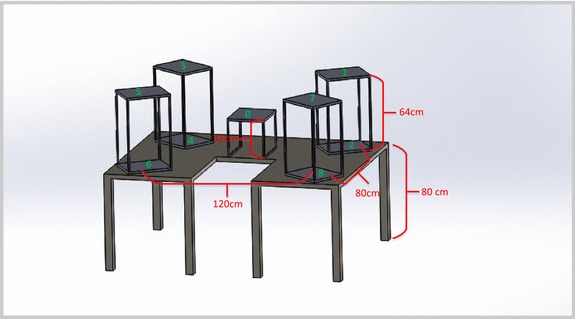
Model of task workspace

**Fig. 5:**
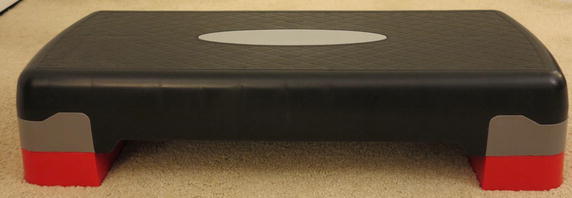
Adjustable platform

**Fig. 6:**
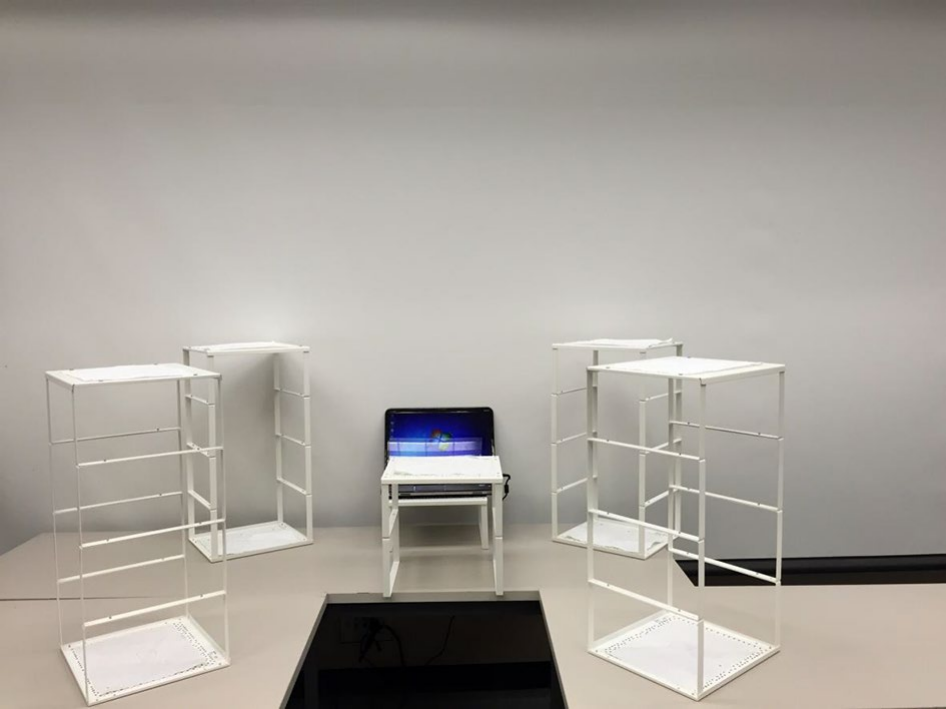
Actual task workspace used for protocol

Figure 4 shows a model of the three-dimensional workspace created for the protocol. The workspace consisted of five shelving units placed on top of a U-shaped table. The U-shaped table was 80 cm tall. Four shelving units, 64 cm tall, were positioned at the four corners of the workspace. The space below the shelving units (i.e. the surface of the table) was accessible. Each shelving unit was used to create two target positions: one at the top of the shelf (height above table = 64 cm) and one at the bottom of the shelf (height above table = 0 cm). This created a total of eight target positions (positions 1–8 in Fig. 4). The fifth shelving unit was 32 cm tall (half the height of the other shelving units) and was placed in the center of the workspace to create the origin position (position 0 in Fig. 4). The participant was asked to stand in the center of the U-shaped table, directly in front of the origin position, and grasp and move the objects to-and-from the various shelving units on the table. The protocol required the top of the target shelves (positions 1, 3, 5 and 7 in Fig. 4) to be 5 cm above the height of the participant’s shoulder, such that, the participant would be required to forward flex his/her shoulder above the horizontal to reach the target positions on top of the shelving unit. In the event that the participant was too short for this constraint, an adjustable platform was provided for the participant to stand on (Fig. 5). The number of shelves and their position relative to the participant was selected to ensure that participants had to move their arms superiorly, inferiorly, medially, laterally, proximally, and distally in order to move objects to-and-from each of the shelves. Figure 6 shows the actual workspace created.

### Grasp types

**Fig. 7:**
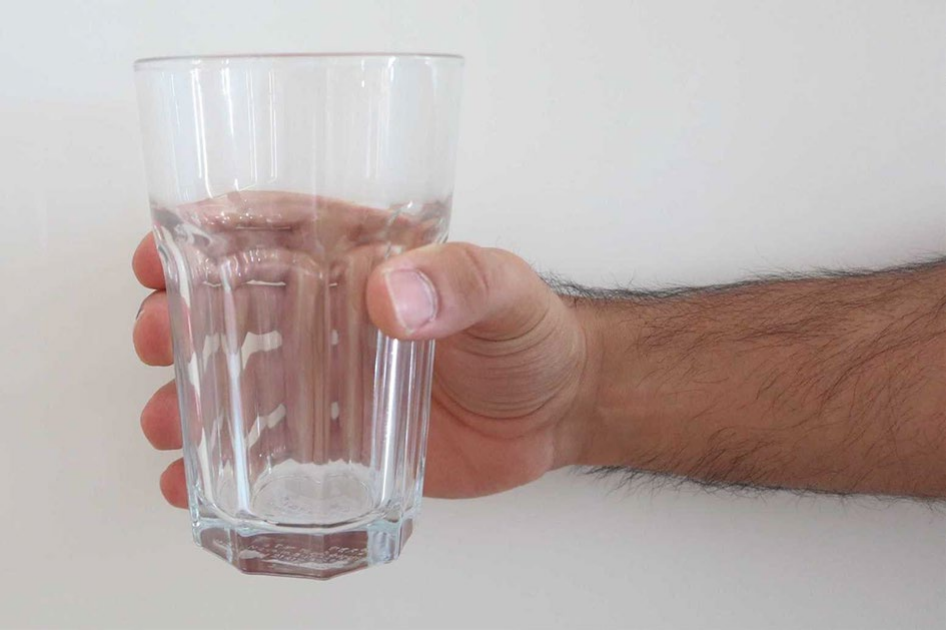
Drinking glass for medium wrap grasp type

**Fig. 8:**
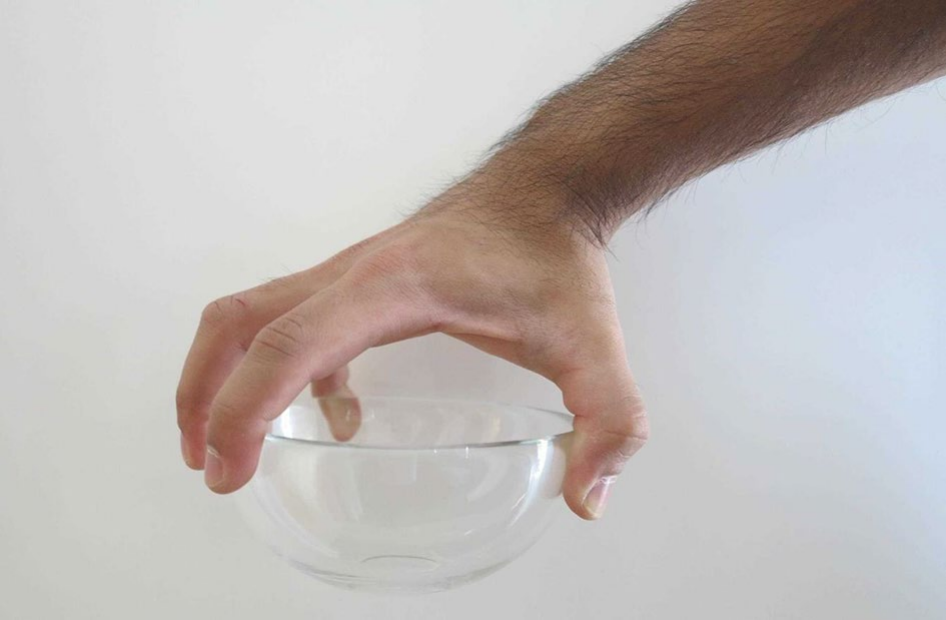
Quarter bowl for precision disk grasp type

**Fig. 9:**
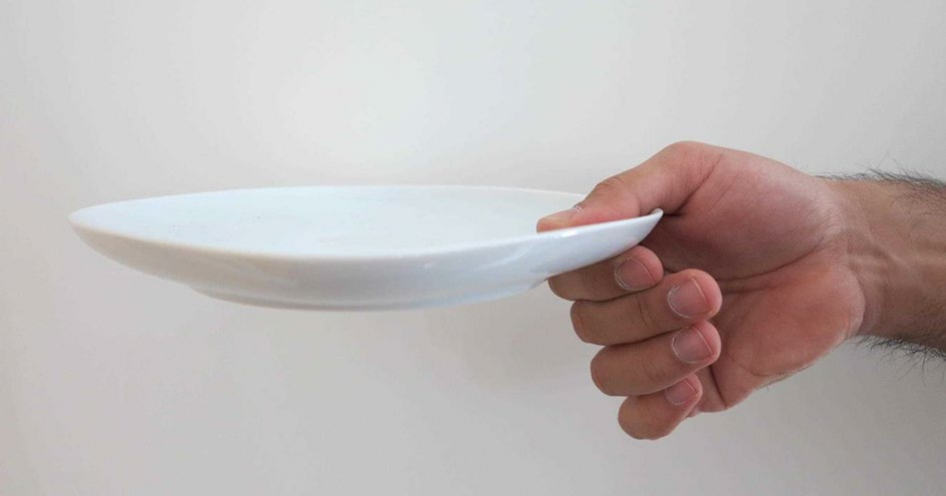
Quarter plate for lateral pinch grasp type

**Fig. 10:**
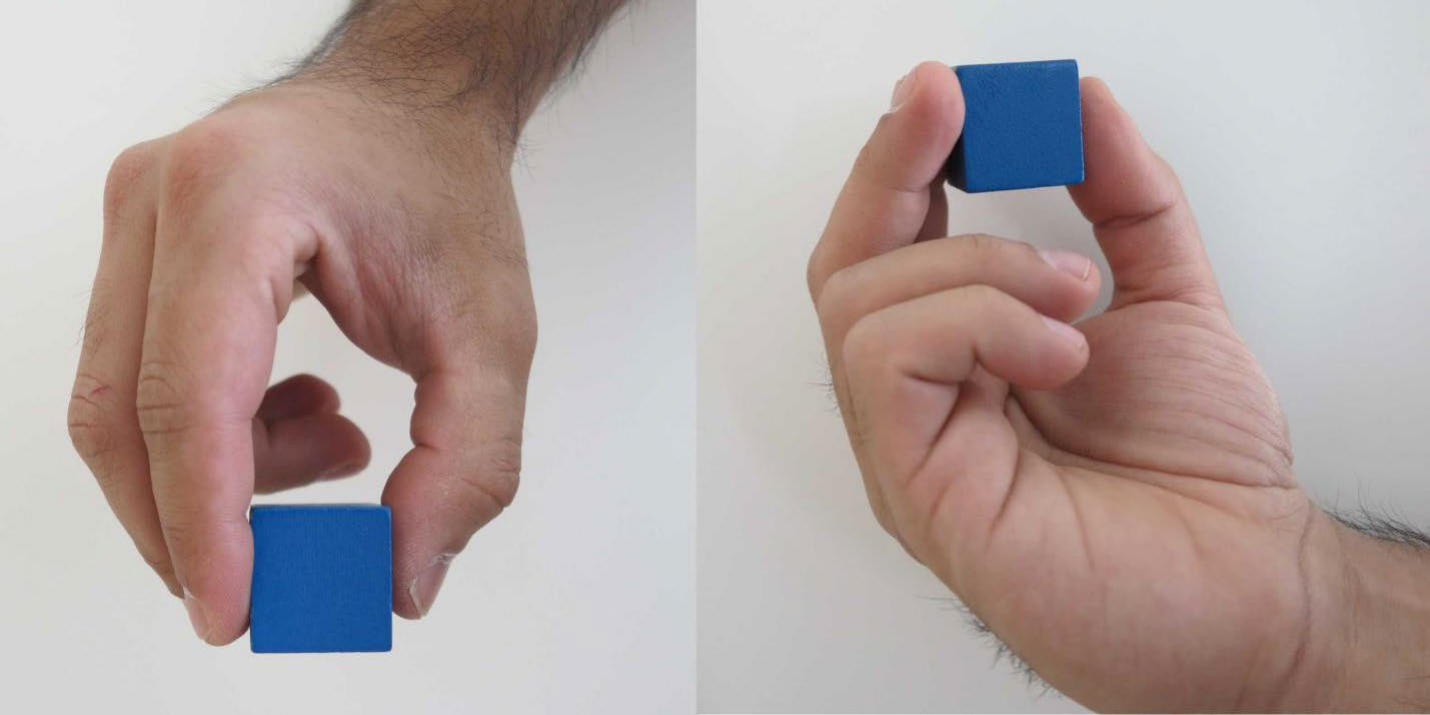
Block for tripod/lateral tripod grasp type

**Fig. 11:**
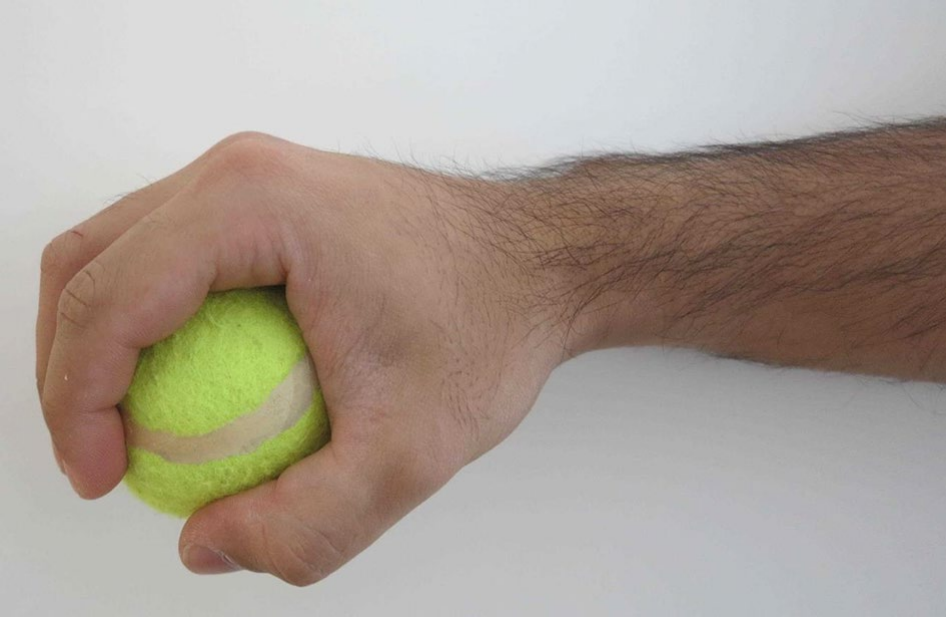
Tennis ball for power sphere grasp type

**Fig. 12:**
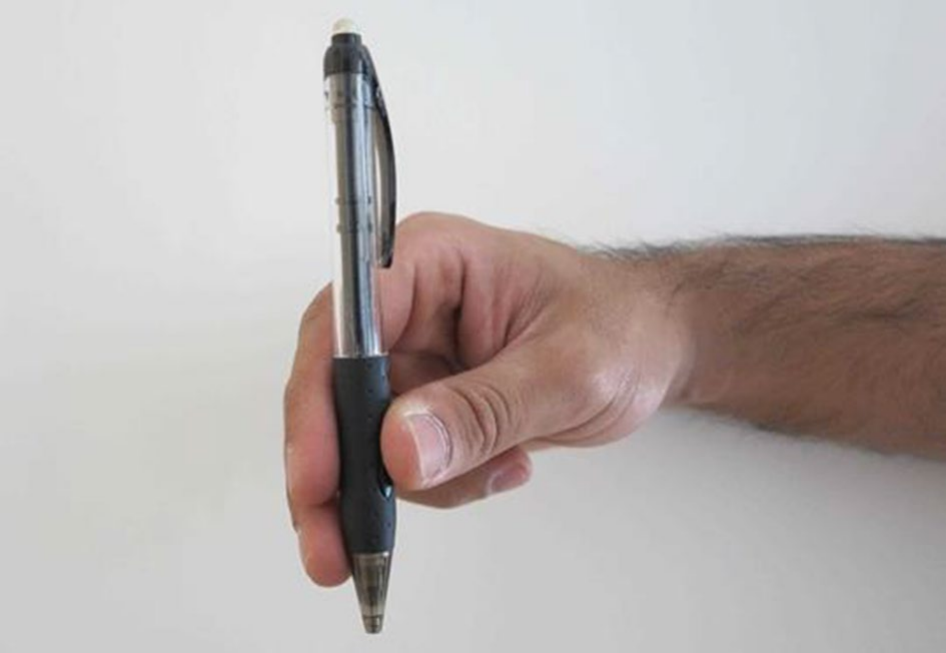
Pen for thumb-2 finger grasp type

Grasp types that are frequently used in ADL were selected for the protocol. Bullock et al. monitored the relative frequency of the different grasp types used by two machinists and two housekeepers as part of their daily activities [12]. We selected seven of the most frequently used grasp types for evaluation: (1) medium wrap, (2) precision disk, (3) lateral pinch, (4) tripod, (5) lateral tripod, (6) power sphere, and (7) thumb-2 finger. Combined, these grasp types account for more than 50% of the grasps that occurred for the two machinists and two housekeepers in Bullock et al.’s study [12]. A specific object was selected for each of the grasp types. The tripod and lateral tripod grasp types were considered identical from the FMG perspective, as they involve identical finger positions and are in fact variations of hand orientation in the three-dimensional workspace. It is noteworthy that other variants of grasp taxonomy do not distinguish between the tripod and lateral tripod grasp types [29]. Table 1 lists the objects selected for each grasp type. Figures 7, 8, 9, 10, 11 and 12 depict the objects selected for each grasp type.

**Table 1:** Objects used for each grasp type evaluated

Index	Grasp type	Object
1	Medium wrap	Drinking glass
2	Precision disk	Quarter bowl
3	Lateral pinch	Quarter plate
4	Tripod/lateral tripod	Block from box and blocks test [30]
5	Power sphere	Tennis ball
6	Thumb-2 finger	Pencil

### Instructions to participants

The protocol consisted of three identical rounds of data collection, each of which consisted of grasp and move activity, and movement without grasping (i.e. with a neutral hand posture). The number of rounds was selected as a compromise between the goals of collecting the maximum amount of data possible, and avoiding muscular and attention fatigue in participants. Participants were asked to use their dominant hands for all activities. Participants started the protocol with their hand in the neutral position (i.e. hand by their side). For grasp and move activity, the participant was asked to move his/her hand to the origin position (position 0 in Fig. 4), grasp and lift an object from the origin position, move and release the object at one of the target positions (positions 1–8 in Fig. 4), and to return his/her hand to the neutral position (by his/her side). Subsequently, the participant was asked to move their hand to the previously used target position (positions 1–8 Fig. 4), grasp and lift the object from the target position (positions 1–8 Fig. 4), and to move and release the object at the origin position, before retuning his/her hand to the neutral position (by his/her side). The participant was asked to repeat this process for each of the target positions starting with position 1 in Fig. 4, and ending with position 8 in Fig. 4. This resulted in sixteen grasp and move actions for each object. It is noteworthy that the movement and placement of the objects at the target locations required the participant to move his/her wrist, elbow, and shoulder joints, and involved arm movement in all three planes of motion. This allowed for the evaluation of the false negative rate (incorrectly predicting a neutral hand posture when a grasp was occurring) of FMG based grasp detection while grasping in the presence of upper-extremity movement.

Data corresponding to movement without grasping were collected in-between the grasp and move activity for each object, in order to evaluate the false positive rate of FMG based grasp detection in the presence of upper-extremity movement. Examples of upper-extremity movement without grasping that may be encountered as part of ADL include raising and putting an arm through a sleeve while donning a garment, waving a hand in greeting, and swinging an arm while walking. Specifically, participants were asked to move their hands to the positions that would correspond to the movement involved in the grasp and move case, but without grasping any object. The participant was asked to move his/her hand from the neutral position, to hover over the origin position (position 0 in Fig. 4), and to then move his/her hand to hover over a target position (positions 1–8 in Fig. 4), before returning his/her hand to the neutral position. The participant was subsequently asked to move his/her hand from the neutral position to the previously used target position (positions 1–8 in Fig. 4), and then to move his/her hand to hover over the origin position (position 0 in Fig. 4), before returning to the neutral position. The sequence was repeated for each target position, resulting in sixteen rounds of movement. The above described movement without grasping, and grasp and move, actions were repeated for each grasp type and object. The entire sequence was repeated three times to form the three rounds of data collection. The instructions used for each round of movement without grasping, and each round of grasp and move activity, with different objects and grasp types, are summarized in Table 2.

**Table 2:** Instructions to participants for each round of movement without grasping and each round of grasp and move activity

Type	Instructions to participant
Movement without grasping	Move from neutral to origin position, and then to target position before returning to neutral. Subsequently move from neutral to target position, and then to origin position before returning to neutral. Repeat across eight target locations, resulting in sixteen movement actions
Grasp and move	Move from neutral to origin position, grasp and pick-up object, move object, place and release object at target position, before returning hand to neutral. Subsequently move from neutral to previous target position, grasp and pick-up object, move object, place and release object at origin position, before returning hand to neutral. Repeat across eight target locations, resulting in sixteen grasp and move actions

### Analysis

#### Features evaluated

Five basic temporal feature extraction techniques were identified for evaluation: (i) mean absolute value (MAV), (ii) root mean squared (RMS), (iii) coefficients of linear fit (LF), (iv) coefficients of parabolic fit (PF), and (v) coefficients of autoregressive model (AR). These feature extraction techniques derive from two of the several ways in which time series can be represented. The MAV and RMS provide a representation of the overall magnitude of the data within the feature window. The LF, PF, and AR are all methods of modelling trends within the data. While more advanced methods of representing time series and extracting temporal features exist, the use of these features allows for clear interpretation of the merits of temporal feature extraction for FMG classification in this preliminary study. The specific rationale for evaluating each of these features is as follows.

MAV is a representation of the overall FMG activity, for the given channel, within the window. The use of an overall representation may lead to a reduction in misclassification due to changes in the FMG signal caused by upper-extremity movement and other outliers in the data. MAV has been used as a feature in the classification of Mechano Myography (MMG) signals [31], and EMG signals [32]. The MAV for each channel can be calculated using (1); where *i* is the sample number from 1 to n, *n* is the window size, and *k* is the channel number from 1 to 16.

1 $$MAV_{k} = \frac{1}{n}\mathop \sum \limits_{i = 1}^{n} \left| {FMG_{k,i} } \right|$$

RMS is a representation of the power of the FMG signal for the given channel, within the window. RMS may relate to grip strength, which could potentially increase the ease of separation of data corresponding to the participant having an object in his/her hand and a neutral hand posture. RMS has been shown to be an effective predictor of force of contraction in MMG signals [33], and has been used in EMG classification [34]. The RMS for each channel can be derived using (2); where *i* is the sample number from 1 to n, *n* is the window size, and *k* is the channel number from 1 to 16.

2 $$RMS_{k} = \sqrt {\frac{1}{n}\left( {FMG_{k,1}^{2} + FMG_{k,2}^{2} + \cdot \cdot \cdot + FMG_{k,n}^{2} } \right)}$$

LF models the trend and amplitude of the FMG for the given channel, within the window. The model may reflect a change in the force exerted by the participant’s musculo-tendinous complex and may capture the temporal change in force that occurs as the user grasps, and releases an object. The LF is part of a wider class of linear model fit features. The use of LF for the preliminary evaluation of temporal features for FMG classification allows for easier interpretation of the utility of linear models. The LF for each channel can be calculated by computationally applying an error minimization routine to the least-squares residual expression in (3) to solve for fit parameters *a* and *b*; where *E* is the residual error term of the fit, *i* is the sample number from 1 to *n*, *n* is the window size, a *k* is the channel number from 1 to 16. In this study, the fitting routine was carried out using the polyfit function in MATLAB [35].

3 $$E_{k} = \mathop \sum \limits_{i = 1}^{n} \left[ {FMG_{k,i} - \left( {a_{k} + b_{k} i} \right)} \right]^{2}$$

PF builds upon the LF and allows for modelling with three degrees of freedom. The additional fit term provided by the PF allows for the modeling of non-linear trends in the data within the window. The model may reflect a change in the force exerted by the participant’s musculo-tendinous complex and may capture the temporal change in force that occurs as the user grasps, and releases an object. The PF has been shown to be an effective feature in handwritten character recognition [36]. The PF is part of a wider class of non-linear model fit features. The use of PF for the preliminary evaluation of temporal features for FMG classification allows for the interpretation of the utility of non-linear models. The PF for each channel can be calculated by computationally applying an error minimization routine to the least-squares residual expression in (4) to solve for fit parameters *a*, *b*, and *c*; where *E* is the residual error term of the fit, *i* is the sample number from 1 to *n*, *n* is the window size, and *k* is the channel number from 1 to 16. In this study, the fitting routine was carried out using the polyfit function in MATLAB [35].

4 $$E_{k} = \mathop \sum \limits_{i = 1}^{n} \left[ {FMG_{k,i} - \left( {a_{k} + b_{k} i + c_{k} i^{2} } \right)} \right]^{2}$$

AR allows for modelling of the linear dependence of a data point on previously occurring data points and a stochastic term. The model may reflect a change in the force exerted by the participant’s musculo-tendinous complex and may capture the temporal change in force that occurs as the user grasps, and releases an object. AR has been shown to be effective for MMG [31] and EMG classification [34]. The AR for each channel can be calculated by computationally solving for the *a*_*i*_ terms in the expression in (5); where *i* is the sample number from 1 to n, *n* is the window size, *k* is the channel number from 1 to 16, and *ɛ* is the stochastic error term. In this study, the order of the autoregressive model was set to be 1 below the number of samples in the window size. The AR parameters were calculated by applying the Yule-Walker method using the aryule function in MATLAB [37].

5 $$FMG_{k,n} = \mathop \sum \limits_{i = 1}^{n - 1} a_{i} FMG_{k,n - i} + \varepsilon$$

**Fig. 13:**
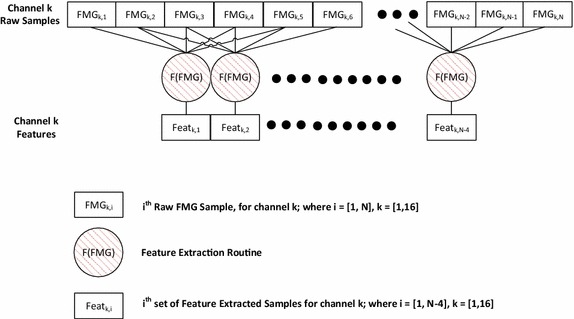
Exemplary 5 Sample, Centre Justified Feature Extraction Scheme. For brevity, only the first two, and last instances of the feature extraction routine are illustrated. *Black dots* are used to indicate additional samples or instances of the feature extraction routine have been omitted from the image. FMG_k,i_ is the ith FMG sample from the kth channel, where k = [1, 16], and i = [1, N]. Feat_k,i_ is the ith set of feature samples for the kth channel, where i = [1, N − 4]

Each feature extraction technique was run on the FMG data using a 0.5 s window (5 samples). In order to evaluate the utility associated with each candidate feature, no additional processing or filtering was conducted on the FMG data before or after feature extraction. The window size was selected based on an empirical analysis of the time-scale in which a transition between a neutral hand posture and a grasp hand posture occurs. In the case of the LF, PF and AR features, the selected window size allows for modelling of the temporal trends (such as slope and curvature) of the FMG signal for a given channel, within a 0.5 s timespan, which would allow for the capture of information on the change in the FMG profile that may occur as the transition to-and-from a grasp posture occurs. In all cases, the feature value associated with a single instantaneous FMG sample for a given channel was found by transforming data around the given sample using a symmetrically centered overlapping window (i.e. feature value corresponding to the current sample depended on the current sample and two samples before and after it). This resulted in an 80% (four out of five samples) overlap between successive feature extraction windows. The feature values were constructed sample-by-sample and channel-by-channel. The feature extraction scheme is depicted in Fig. 13.

#### Feature evaluation framework

A feature evaluation framework was developed in MATLAB [38] in order to be able to evaluate the off-line classification performance obtained with different features of the FMG signal. The framework was based on the wrapper feature evaluation method [39–41]. In the wrapper method, a classifier is trained and tested using each candidate feature in order to understand their individual effectiveness for classification purposes. In this study, the wrapper method was used to evaluate off-line classification performance with each of the features and the raw FMG signal. While other more computationally efficient methods for feature evaluation and selection exist, the wrapper method was chosen as it allows for unambiguous understanding of the effectiveness of each feature proposed [39]. The effectiveness of each feature was evaluated using the area under the receiver operating curve (AUC). Receiver operating curves plot the true positive rate (sensitivity) against the false positive rate (1-specificity) for a classifier [42]. The ideal classifier will have a true positive rate of 1 (i.e. sensitive) and a false positive rate of 0 (i.e. specific), yielding an AUC of 1. The classification accuracy (i.e. ratio of the number of correctly predicted samples to the total number of samples) was also calculated for each feature, but was not used as a primary measure of the effectiveness of candidate features. AUC was chosen as the measure of effectiveness as it is insensitive to the relative distribution of the classes within the data, and hence, provides a robust method of evaluating classification performance when compared to classification accuracy [42].

**Fig. 14:**
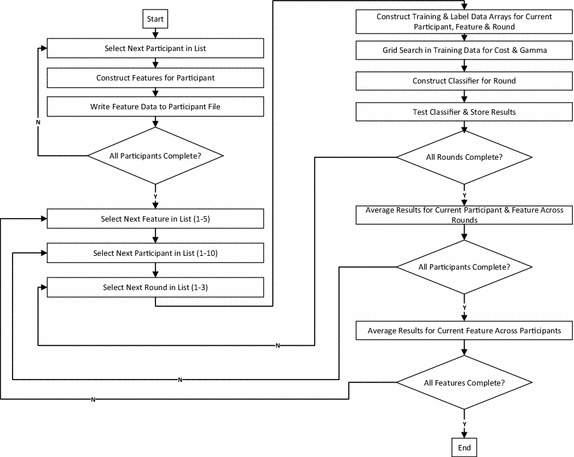
Feature evaluation flowchart

The feature evaluation framework consisted of two run-time stages, as depicted in Fig. 14. In the first stage, features were extracted for all three rounds of data collection for each participant and stored to file for future classification. In the second stage, classification performance with each of the extracted features was evaluated for each participant. A support vector machine (SVM) with a radial basis function kernel (RBF), implemented via the LIB-SVM library [43] for MATLAB was used for classification. AUC and accuracy were evaluated in a three fold cross-validation scheme. Specifically, data from a given round was classified using a classifier that was constructed with data from the remaining two rounds for the participant. For each round, a ten fold cross-validation scheme within the training data was used to determine the optimal cost and gamma for the RBF-SVM via a grid search. The average AUC and accuracy obtained across three folds for a given feature and participant was calculated, and subsequently the average AUCs and accuracies across all participants were calculated for each feature. Features that resulted in increased AUC when compared to the raw FMG signal were identified. A single-tailed, paired samples Student’s t test with alpha of 0.05 was used to determine if the increase in AUC seen due to the classification of a particular feature, in comparison to the classification of the raw FMG signal, was statistically significant.

## Results

### Participant information

Ten healthy participants were recruited for this study. Participant age, gender, and the need for the height adjusting step to meet the protocol’s height restriction, are listed in Table 3.

**Table 3:** Participant information

ID	Age (years)	Gender	Height adjusting step required
1	23	Female	Yes
2	22	Female	No
3	27	Female	Yes
4	21	Male	No
5	23	Male	No
6	23	Male	No
7	24	Male	No
8	25	Female	Yes
9	21	Male	No
10	27	Male	No

### Feature evaluation results

**Fig. 15:**
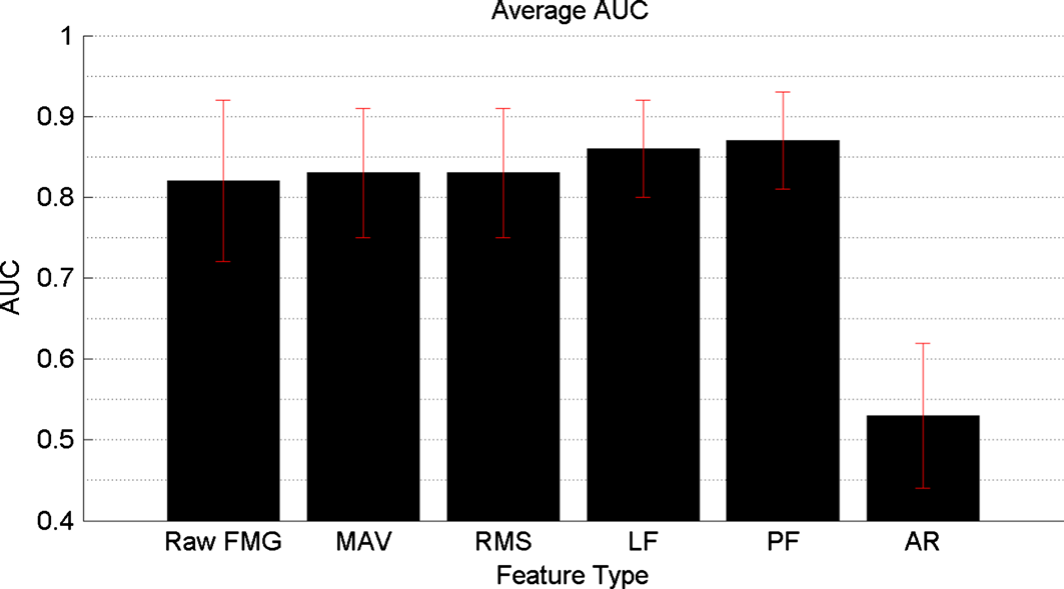
AUC for all features evaluated

Figure 15 depicts the AUC obtained for each feature evaluated and the raw FMG signal. Four of the five features evaluated resulted in modest increases in AUC when compared to the raw FMG signal; two features (LF and PF) produced statistically significant increases in AUC. The AUC, accuracies, and associated standard deviations for the four features that demonstrated increased performance are listed in Table 4. Table 4 also lists the P values (single-tailed, paired samples Student’s t test) associated with the AUC for each feature in relation to the AUC associated with the raw FMG signal. The raw FMG signal yielded an AUC of 0.82 ± 0.10 and an accuracy of 88.79 ± 5.18%. The largest increase in AUC over the raw FMG signal was obtained using PF, yielding an AUC of 0.869 ± 0.061 and an accuracy of 90.64 ± 4.30%. The increases in AUC (P = 0.011 < 0.05) and accuracy (P = 0.031 < 0.05) observed over the raw FMG signal are statistically significant for PF.

**Table 4:** Features with increased AUC over the raw FMG signal

Feature	AUC		Accuracy		P value
	Value	σ	Value (%)	σ (%)	
PF	0.869	0.061	90.635	4.298	0.011
LF	0.862	0.065	89.859	4.940	0.017
MAV	0.834	0.076	86.703	6.672	0.152
RMS	0.829	0.075	86.909	6.730	0.253
Raw FMG	0.819	0.098	88.792	5.178	N/A

## Discussion

The objective of this study was to explore the utility of classifying temporal features of the FMG signal for FMG data associated with a variety of grasp-types, in the presence of upper-extremity movements, for the two-class grasp detection problem of grasp, regardless of grasp type, versus no grasp (i.e. no object in hand). The MAV, RMS, LF, and PF features all yielded modest increases in AUC when compared to the raw FMG. However, increases seen for the MAV and RMS were not statistically significant (single-tailed, paired samples Student’s t test; P = 0.078). In contrast, LF and PF features yielded larger and statistically significant increases in performance. A potential explanation for the superior performance of the LF and PF features is the fact that they attempt to model the trends within the data window, while the MAV and RMS both calculate an estimate of the overall magnitude of the data for the given window. It is possible that the capturing of trend information allows for greater ease of separation of FMG data when compared to capturing only magnitude information. The capturing of trend information may reflect temporal changes in the FMG signal that occur when a transition into, or out of a grasp occurs. Furthermore, the trending of FMG data may also provide increased robustness to outliers, or noise in the data, as a single transient change in the signal will be attenuated by the remaining samples in the feature extraction window. It is noteworthy that in order to allow for the evaluation of the utility of the candidate features, all analysis steps in this study used the raw FMG data without any filtering. While the use of frequency selective filters will not enable the modeling of trend information within a window of data, it is possible that filtering the raw FMG signals could also yield increased robustness to noise and outliers in the FMG data.

These results suggest that the use of feature extraction techniques that attempt to model the FMG data as a linear, or non-linear time series may yield modest increases in grasp detection performance, when compared to feature extraction techniques that attempt to capture an overview of the FMG data within the window, and when compared to the classification of raw FMG data. It is noteworthy however, that the use of model-based features result in additional feature value(s) for each sample of data on each FMG channel, which in turn increases the computational cost of classification. For instance, the LF feature extraction step produces two feature samples for each window of the FMG signal evaluated. The benefits obtained by using this class of features will have to be traded off with additional computational time and complexity for the different applications of FMG based grasp detection. It may also be possible to reduce the amount of overlap between successive feature extraction windows in order to reduce the size of data, and associated computational requirements for classification of this class of features. The use of an additional fitting term in the PF did not significantly improve classification performance over the LF despite the additional feature value for each sample of data on each FMG channel. This indicates that the use of non-linear models may not provide benefit to offset the additional computation cost when compared to linear models. It is also possible that the PF feature resulted in over fitting of the data as the feature extraction window size was held constant despite the increased fitting term provided by the PF. We acknowledge that the use of a fixed, 5-sample window is a limitation of this study. To overcome this limitation, future work should explore the effect of varying feature extraction window sizes, symmetries, and overlaps.

Despite the noted advantages obtained via the LF and PF features, the use of the AR feature resulted in poor classification performance. The AR calculation models an invariant mean, stationary process [37]. A potential explanation for this finding is that the stationarity assumption may not hold for the FMG data collected in this study. The mean value of the FMG signal may drift over time as the band slackens or tightens on the user’s musculature. The use of models that account for a shifting mean or baseline such as the autoregressive moving average model may provide increased utility in FMG based grasp detection applications. It is also possible that orders of the AR model selected for the window configuration in this study were inappropriate for the FMG data collected.

## Future work

The scope of this study was limited to a preliminary evaluation of the utility of classifying temporal features of the FMG signal for the two-class FMG grasp detection of grasp, regardless of grasp type, versus no grasp (i.e. no object in hand). The study focused on the evaluation of two types of basic temporal feature extraction methods in healthy volunteers. The results indicate that features that model temporal trends within the data may increase FMG based grasp detection performance. Based on these promising results, future work should include a focused evaluation of additional features that model temporal trends within the FMG data for grasp classification. Furthermore, the effect of varying feature extraction window sizes, symmetries, and overlaps should also be evaluated. Finally, the use of model-based temporal features should be evaluated with FMG data from individuals with stroke, who might ultimately benefit from this technology.

## Conclusion

In this study, the utility of classifying temporal features of the FMG signal for the two-class grasp detection problem of grasp, regardless of grasp type, versus no grasp (i.e. no object in hand) was evaluated. An experimental protocol consisting of multiple repetitions of grasp and move tasks that are commonly used in ADL was designed. The protocol also included movement without grasping in order to evaluate the robustness of FMG based grasp detection to false positives. The use of MAV, RMS, LF, and PF features yielded modest improvements in grasp detection performance. However, increases in classification performance seen for MAV and RMS were not statistically significant. The largest increase in classification performance over the raw FMG signal was obtained for PF, corresponding to a relative increase in AUC of 6.1%. The LF also provided a statistically significant increase in classification performance. These results suggest that using features that model trends in data may provide increased FMG based grasp detection performance when compared to using the raw FMG signal, and when compared to using feature extraction techniques that attempt to capture an overview of FMG data within the window. The changes in performance between LF and PF were not statistically significant, indicating that the use of non-linear models may not provide benefit to offset the additional computation cost when compared to linear models. These results pave way for further evaluation of model-based temporal features of the FMG signal with varying window configurations, and an evaluation of the utility of classifying features of the FMG signal from individuals with stroke, such that FMG may be eventually used for grasp detection in stroke rehabilitation applications.

## Abbreviations

ADL: activities of daily living
AR: autoregressive model
AUC: area under the receiver operating curve
EMG: electromyography
FMG: Force Myography
LF: linear fit
MAV: mean absolute value
MMG: Mechano Myography
PF: parabolic fit
RBF: radial basis function
RMS: root mean squared
SVM: support vector machine

## References

[1] Wininger M, Kim N-H, Craelius W (2008). Pressure signature of forearm as predictor of grip force. J Rehabil Res Dev.

[2] Castellini C, Ravindra V. A wearable low-cost device based upon force-sensing resistors to detect single-finger forces. In: 5th IEEE RAS & EMBC international conference on biomedical robotics and biomechatronics, Sao Paulo, Brazil. 2014.

[3] Dementyev A, Paradiso JA. WristFlex: low-power gesture input with wrist-worn pressure sensors. In: Proceedings of the 27th annual ACM symposium on user interface software and technology. 2014. p. 161–6.

[4] Sadarangani G, Menon C. A wearable sensor system for rehabilitation applications. In: IEEE international conference on rehabilitation robotics, Singapore. 2015.

[5] Xiao ZG, Menon C (2014). Towards the development of a wearable feedback system for monitoring the activities of the upper-extremities. J Neuroeng Rehabil.

[6] Cho E, Chen R, Merhi L-K, Xiao Z, Pousett B, Menon C (2016). Force Myography to control robotic upper extremity prostheses: a feasibility study. Front. Bioeng. Biotechnol..

[7] Sakr M, Menon C. On the estimation of isometric wrist/forearm torque about three axes using Force Myography. In: IEEE RAS/EMBS international conference on biomedical robotics and biomechatronics, Singapore. 2016.

[8] Nudo R, Wise B, SiFuentes F, Milliken G (1996). Neural substrates for the effects of rehabilitative training on motor recovery after ischemic infarct. Science.

[9] Murata Y, Higo N, Oishi T, Yamashita A, Matsuda K, Hayashi M, Yamane S (2008). Effects of motor training on the recovery of manual dexterity after primary motor cortex lesion in macaque monkeys. J Neurophysiol.

[10] Dobkin BH (2004). Strategies for stroke rehabilitation. Lancet Neurol.

[11] Gresham GE, Alexander D, Bishop DS, Giuliani C, Goldberg G, Holland A, Kelly-Hayes M, Linn RT, Roth EJ, Stason WB, Trombly CA (1997). Rehabilitation. Stroke.

[12] Bullock IM, Zheng JZ, Rosa SDL, Guertler C, Dollar AM (2013). Grasp frequency and usage in daily household and machine shop tasks. IEEE Trans Haptics.

[13] Yap HK, Mao A, Goh JCH, Yeow C-H. Design of a wearable FMG sensing system for user intent detection during hand rehabilitation with a soft robotic glove. In: IEEE international conference on biomedical robotics and biomechatronics, Singapore. 2016.

[14] Kadkhodayan A, Jiang X, Menon CJ (2016). Continuous predicting finger movements using Force MyoGraphy. Med. Biol. Eng..

[15] Ambar RB, Poad HB, Ali AM, Ahmad MS, Jamil MM. Multi-sensor arm rehabilitation monitoring device. In: International conference on biomedical engineering, Penang. 2012.

[16] Yungher DA, Wininger MT, Barr JB, Craelius W, Threlkeld AJ (2011). Surface muscle pressure as a measure of active and passive behavior of muscles during gait. Med Eng Phys.

[17] Natarajan GS, Winninger M, Kim NH, Craelius W (2012). Relating biceps EMG to elbow kinematics during self-paced arm flexions. Med Eng Phys.

[18] Li N, Yang D, Jiang L, Liu H, Cai H (2012). Combined use of FSR sensor array and SVM classifier for finger motion recognition based on pressure distribution map. J Bionic Eng.

[19] Yngher D, Craelius W. Discriminating 6 grasps using Force Myography of the forearm. In: BMES annual fall meeting. 2006.

[20] MacKenzie CL, Iberall T (1994). The grasping hand.

[21] Zardoshti-Kermani M, Wheeler B, Badier K, Hashemi R (1995). Classification of the myoelectric signal using time-frequency based representation. Med Eng.

[22] Fulcher BD, Jones NS (2014). Highly comparative feature-based time-series classification. IEEE Trans Knowl Data Eng.

[23] InterlinkElectronics. FSR^®^ integration guide & evaluation parts catalog with suggested electrical interfaces. 2010.

[24] Jiang X, Merhi L-K, Menon C. Force exertion affects grasp classification using Force Myography. IEEE Trans Human-Machine Syst. 2017;(99):1–8. doi:10.1109/THMS.2017.2693245.

[25] Amft O, Junker H, Lukowicz P, Tröster G, Schuster C. Sensing muscle activities with body-worn sensors. In: International workshop on wearable and implantable body sensor networks (BSN’06), Cambridge, MA. 2006.

[26] Heldman DA, Jankovic J, Vaillancourt DE, Prodoehl J, Elble RJ, Giuffrida JP (2011). Essential tremor quantification during activities of daily living. Parkinsonism Relat Disord.

[27] Xiong Y, Quek F (2006). Hand motion gesture frequency properties and multimodal discourse analysis. Int J Comput Vis.

[28] National Instruments. LabVIEW System Design Software. National Instruments, (Online). http://www.ni.com/labview/. Accessed 19 Jan 2016.

[29] Cutkosky MR (1989). On grasp choice, grasp models, and the design of hands for manufacturing tasks. IEEE Trans Robot Autom.

[30] Mathiowetz V, Volland G, Kashman N, Weber K (1985). Adult norms for the box and blocks test of manual dexterity. Am J Occup Ther.

[31] Alves N, Chau T. Recognition of forearm muscle activity by continuous classification of multi-site mechanomyogram signals. In: International conference of the IEEE EMBS, Buenos Aires. 2010. 10.1109/IEMBS.2010.5627754 21097038

[32] Hudgins B, Parker P, Scott RN (1993). A new strategy for multifunction myoelectric control. IEEE Trans Biomed Eng.

[33] Madeleine P, Bajaj P, Søgaard K, Ardengt-Nielsen L (2001). Mechanomyography and electromyography force relationships during concentric isometric eccentric contractions. J Electromyogr Kinesiol.

[34] Khokhar ZO, Xiao ZG, Menon C (2010). Surface EMG pattern recognition for real-time control of a wrist exoskeleton. Biomed Eng Online.

[35] MathWorks. Polyfit, MathWorks, (Online). http://www.mathworks.com/help/matlab/ref/polyfit.html. Accessed 05 June 2016.

[36] Kumar M, Jundal MK, Sharma RK. Weka-based classification techniques for offline handwritten Gurmukhi character recognition. In: International conference on soft computing for problem solving. 2014.

[37] MathWorks. aryule, MathWorks, (Online). http://www.mathworks.com/help/signal/ref/aryule.html. Accessed 21 May 2016.

[38] MathWorks. http://www.mathworks.com. MathWorks, (Online). http://www.mathworks.com/products/matlab/. Accessed 16 Jan 2016.

[39] Guyon I, Elisseeff A (2003). An introduction to variable and feature selection. J Mach Learn Res.

[40] Kohavi R, John GH (1997). Wrappers for feature subset selection. Artif Intell.

[41] Caruana R, Freitag D. Greedy attribute selection. In: International conference on machine learning. 1994. p. 28–36.

[42] Bradley AP (1997). The use of the area under the ROC curve in the evaluation of machine learning algorithms. Pattern Recogn.

[43] Chang C-C, Lin CJ (2011). LIBSVM: a library for support vector machines. ACM Trans Intell Syst Technol.

